# User evaluation of a virtual reality simulation for anaesthesiologists-intensivists for non-technical skills^☆^

**DOI:** 10.1016/j.pecinn.2025.100441

**Published:** 2025-10-16

**Authors:** Krista Hoek, Monique van Velzen, Elise Sarton, Christina Jaschinski

**Affiliations:** a Department of Anaesthesiology, LUMC, Leiden, the Netherlands; b Department of Anaesthesiology, Amsterdam Medical Center, the Netherlands; c Research Group Technology, Health & Care and Saxion Extended Reality Lab, Saxion University of Applied Sciences, the Netherlands

**Keywords:** VR simulation, Immersive learning, Immersive VR, Proteus effect, Medical education, Crew resource management, Kirkpatrick, Health communication skills, Virtual Reality

## Abstract

**Objective:**

Simulation training in medical settings is a cornerstone in medical education to practice technical and non-technical skills. The objective of this study was to evaluate user experiences and initial reactions to a multiplayer virtual reality (VR) simulation designed for anaesthesiologists.

**Methods:**

A user-centred design approach guided the development of a VR-scenario training simulating a case of massive blood loss. Seven anaesthesiology residents and trainers from a Dutch academic hospital participated in qualitative and quantitative evaluations. Quantitative measures included questionnaires on presence, agency, physical dyscomfort, and perceived training value. Qualitative data were collected through structured observations and interviews.

**Results:**

Participants reported a high sense of physical presence and moderate agency, with lower levels of self-presence. The VR environment was perceived as realistic and engaging. Challenges included unfamiliarity with VR controls, abstract avatars, and limited haptic feedback. Despite these barriers, participants recognized the potential of VR for team-based learning of non-technical skills, especially with prior onboarding or practice sessions.

**Innovation:**

This study introduces a novel application of multiplayer VR simulation in anaesthesiology training, focusing on non-technical skills. The integration of user-centred design with qualitative and quantitative evaluation provides insights into the feasibility and acceptability of VR as an educational tool in this context.

**Conclusions:**

VR simulation shows promise for training non-technical skills in anaesthesiology. While participants appreciated the realism and collaborative aspects, enhancements in usability and interaction design are necessary for broader implementation and impact.

## Highlights

• VR simulation was developed to train non-technical skills in anaesthesiology.
• Realistic OR design and multiplayer features enhanced immersion.
• Users reported high presence but struggled with interaction controls.
• Abstract avatars and limited feedback reduced social presence.

## Graphical abstract

**Figure:**
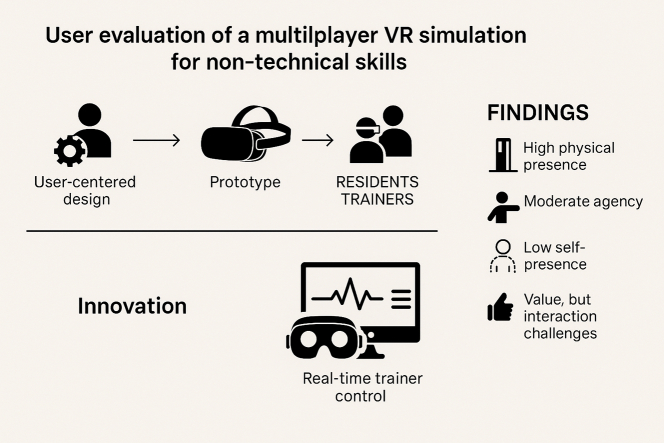

## 1 Introduction

A fundamental aspect of medical education involves training through realistic clinical scenarios that enable healthcare professionals to develop their skills in a structured and safe learning environment. [1,2]. Simulation-based learning creates a controlled environment where healthcare providers can safely practice responding to medical emergencies or rare complications, allowing them to gain valuable experience without risking patient safety.

For anaesthesiologists and intensivists, managing an acutely deteriorating patient demands a combination of technical and non-technical skills in high-pressure situations. These skills include procedures like tracheal intubation, airway management, and vascular access, along with crucial medical knowledge for diagnosing, dosing, and decision-making. Moreover, crew resource management (CRM), situational awareness, communication, and leadership are essential components of effective teamwork. Multitiered rapid response systems can significantly improve patient outcomes by coordinating timely and efficient interventions in critical moments.

In medical education, skill acquisition can be guided by the work of Cutrer (2017) who developed a conceptual model of Master Adaptive Learners [3]. According to this model, clinical decision making may involve *routine expertise* based on specific knowledge and skills an expert has learned over time. On the other hand, *adaptive expertise* may be needed when “routine approach” is not working, and new concepts and solutions need to be sought. The mastery of both types of expertise creates an optimal adaptability corridor, necessary for optimal patient care.

Indeed, medical competency is constructed in social contexts and trainees need to be active learners rather than passive recipients of knowledge [2]. While several studies have shown the effectiveness of simulation-based training over the last decade, increasing pressure on budget and logistic limitations, need for alternative methods of simulation have emerged. Many current simulation programs lack the ability to adapt scenarios in real-time, limiting their effectiveness in creating immersive training environments. Real-time adaptability is essential for providing personalized learning experiences that respond dynamically to the trainee's actions, allowing for a broader range of scenarios and deeper engagement in the training process [1].

Immersive virtual reality (VR) uses a head-mounted display (HMD) to fully engage users by isolating them from the outside world. It offers flexible, cost-effective training opportunities that can be accessed anywhere, enhancing learning experiences without the limitations of location or time [4]. Artificial intelligence is used to visualise dynamic relationships in complex virtual environments. Immersive VR can produce a visceral feeling of being in a simulated world, a form of spatial immersion called *Presence* [5]. Also, in virtual interactions, participant's avatars can affect their attitude, perception, and behaviour in a conscious or unconscious matter known as the *Proteus Effect* [6,7]. A magnetic sensor inside the HMD feeds the magnetic sensor by informing about head or eye movements which changes the displayed graphics. However, there are also potential drawbacks such as limited haptic (tactile) feedback [8] and the absence of non-verbal cues in the trainees' digital avatars [4]. How, and to what extent this influences the learning of communication skills is not known. Importantly, there is limited evidence on how anaesthesiologists themselves perceive and experience VR-based training, especially about communication and teamwork. Understanding their reactions is essential before broader implementation, since user acceptance will determine whether VR can realistically complement traditional simulation. This study therefore addresses a gap by systematically evaluating anaesthesiologists' initial experiences with a prototype VR training program.

**Fig. 1:**
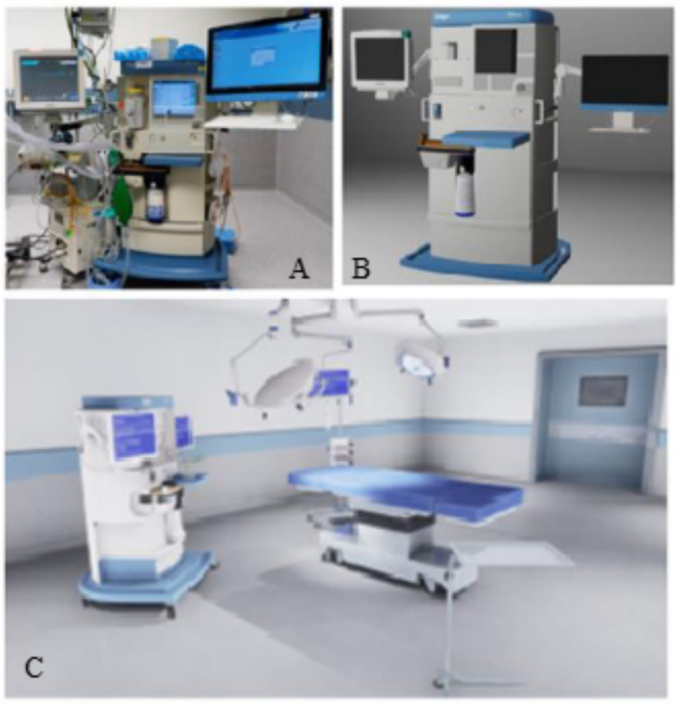
View of a photo (A), the digital design (B), and the incorporation in the virtual OR environment (C).

In earlier work, we have described the feasibility and development of an adaptive VR-scenario training program for anaesthesiologists-intensivists [9]. Key features of this VR-scenario training program include real-time adaptability of patient vital signs, a multiplayer environment for team-based crew management, trainer-controlled scenario adjustments during sessions as can be seen in Fig. 1.

**Fig. 2:**
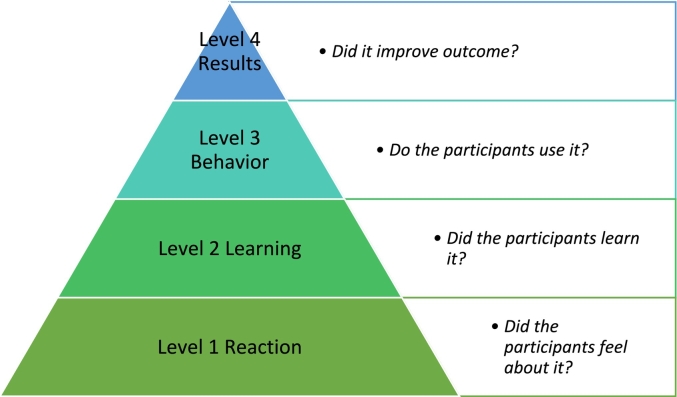
Kirkpatrick's four-level training evaluation model.

In this paper we describe the evaluation of our VR-scenario training program prototype, focusing primarily on Level 1 (Reaction) of Kirkpatrick's four-level training evaluation model as illustrated in Fig. 2 [10,11]. Specifically, we assessed participants' immediate reactions to the VR-scenario training program. Our secondary objectives also align with Level 1, as they included evaluating the perceived likability of the VR training and identifying any side effects experienced during its use. As a tertiary objective, we gathered participant recommendations for improving the prototype.

## 2 Methods

This research was carried out by the LUMC, a tertiary academic hospital in Leiden, the Netherlands and Saxion University of Applied Sciences. The protocol was approved by the Institutional Science Committee of the Anaesthesiology science department and obtained a waiver from the Institutional Review Board (NWMO-LUMC) and underwent independent peer-review from the Tech For Future funding body.^1^ Informed consent was obtained prior to inclusion, participation was voluntary, and privacy rights were aligned with the Declaration of Helsinki and GDPR guidelines.

### 2.1 User-centred design

The field of Human Factors and Ergonomics (HFE) focuses on understanding the interaction between humans and systems in order to design products, processes or systems to complement human behaviour in order to improve human well-being and overall system performance [12]. User-centred design (UCD) is a commonly used methodological approach in HFE to ensure that user's needs and preferences are at the core of the design process [13]. UCD is an iterative process that involves users throughout the design process through a variety of quantitative and qualitative methods and tools. The UCD cycle consists of different stages starting with an in-depths analyses of the user' context and needs which directly inform the design requirements. This is typically followed by iterative design and evaluation of concepts, initial prototypes and later high-fidelity prototypes [14]. In the implementation stage the final prototype is translated into a functional product and tested to ensure that the design meets the requirements. After releasing a product to the user, performance and user feedback is continuously monitored to conduct further improvements and ensure the product continues to meet user's needs [15].

**Fig. 3:**
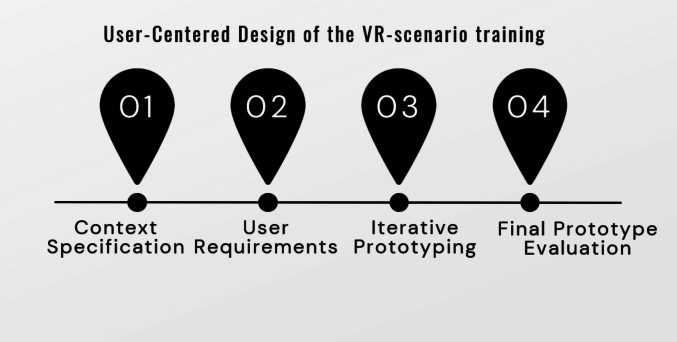
User-centred design approach of the VR-scenario training.

For the development of the VR-scenario training we employed a UCD approach involving simulation trainers and residents from the anaesthesiology department as our end-users. Our complete UCD approach is shown in Fig. 3. The current paper focuses on the evaluation of the final prototype. The analysis of the context and user requirements will be published elsewhere. The iterative development process was described in a previous publication (Hoek et al. 2024).

### 2.2 Description of the final prototype

The VR prototype was developed using the Unreal Engine for use on Meta Quest 2 headsets. The current version presents a segment of a massive blood loss scenario, focusing on essential medical tasks such as applying standard anaesthesia vital monitoring, administering bolus and continuous infusion medication, and initiating fluid and blood transfusions. The virtual operating room closely mirrors the real OR environment at the participating hospital, with equipment and layout modelled in detail. Users navigate the scenario in a multiplayer setting using abstract avatars, while the patient avatar displays high-fidelity visual features. During the simulation, trainers can adjust physiological parameters—including blood pressure, oxygen saturation, haemoglobin levels, skin appearance (rash), and pupil dilation—in real time via a dedicated control dashboard, allowing for dynamic scenario management.

### 2.3 User evaluation of the final VR-scenario training prototype

**Fig. 4:**
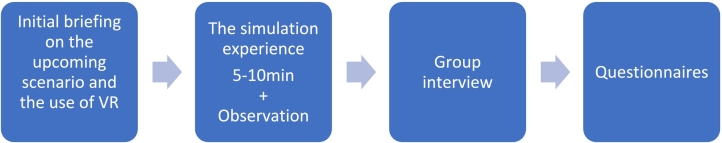
Schematic overview of the procedure of user evaluation study.

The user evaluation of the final prototype of the VR-scenario training was conducted at LUMC. We used a combination of qualitative and quantitative measurements to assess the initial user experience and reaction to the VR-scenario training (Kirkpatrick's level 1) as shown in Fig. 4. The quantitative data was used to get an objective impression of the user experience and served as a quantified reference point to capture the progression of user experience through different design iterations. The qualitative data provided more in-depth information about the user experience and helped to contextualize quantitative scores.

#### 2.3.1 Participants

Residents and simulation trainers who worked at the LUMC hospital were invited by mail to participate. Participants with physical incapacity to use VR were excluded. Participants did not receive financial compensation.

#### 2.3.2 Procedure

Each test session began with a pre-briefing about the equipment, learning objectives and the upcoming scenario and would last up to 10 min. Up to 3 participants were testing the VR-scenario training at the same time to evaluate the multi-user feature. During the simulation experience an observation protocol was completed. After the training session each test group was interviewed about their experience. Subsequently participants were provided with a link to a user experience questionnaire which they could complete at their convenience.

#### 2.3.3 Measurements

The observation protocol included ten open-ended questions focused on user interaction and comfort, such as whether participants experienced difficulties handling the controllers or signs of physical discomfort (e.g. nausea or dizziness). The post-session questionnaire combined validated tools and custom items. Presence was assessed using the 15-item Multimodal Presence Scale (MPS) [16], which measures physical, social, and self-presence [17]. Agency—participants' sense of control over their virtual body—was measured using the agency subscale of the Virtual Embodiment Questionnaire (VEQ) [18], consisting of four items. Additional self-developed items explored physical discomfort and the perceived value of VR compared to traditional mannequin-based training. Responses were collected using a 5-point Likert scale, except for the VEQ agency items, which used the original 7-point scale. An overview of the instruments is presented in Table 1.

**Table 1:** Overview of the quantitative measurements.

Scale	No. of items	Example Item	Response scale	Source
MPS-physical presence	5	While I was in the virtual environment, I had a sense of “being there”.	5 point Likert scale (completely disagree – completely agree	[16]
MPS-social presence	5	I felt like I was in the presence of another person in the virtual environment.	5 point Likert scale (completely disagree – completely agree	[16]
MPS-self-presence	5	I felt like my virtual embodiment was an extension of my real body within the virtual environment	5 point Likert scale (completely disagree – completely agree	[16]
VEQ-agency	4	I felt as if I was controlling the movement of the virtual body.	7 point Likert scale (completely disagree – completely agree	(Roth & Latoschik, 2020)
Physical discomfort	3	I experienced nausea while working in the VR-environment	5 point Likert scale (completely disagree – completely agree	*Self-developed*
Perceived value	2	Once I am experienced in working in virtual reality, training simulations in the virtual environment would be a good alternative to physical simulations with a mannequin.	5 point Likert scale (completely disagree – completely agree	*Self-developed*

The interview lasted approximately 20 min and consisted of three parts. First, participants were asked about their general experience with the VR-scenario, including enjoyment, hardware interaction, discomfort, and perceptions of presence and agency. The second part focused on specific features, such as administering IVs and using the anaesthesia machine. Finally, participants reflected perceived value for training non-technical skills in a simulation context. Participants were also invited to share their ideas on further development of the prototype.

### 2.4 Data analysis

Quantitative data were analysed using descriptive statistics in IBM SPSS, focusing on physical presence, social presence, self-presence, agency, physical discomfort, and perceived value. Due to the small sample size, no inferential tests were conducted. Thematic analysis was used to examine qualitative data [19]. Observations and interviews were reviewed for recurring patterns related to user experience, presence, agency, and perceived training value. Themes were identified by one researcher (CJ), and illustrative quotes were selected. We collected both quantitative and qualitative data, which were analysed separately and considered with equal importance to provide complementary insights.

## 3 Results

### 3.1 Sample characteristics

Seven anaesthesiology professionals from LUMC participated in the user evaluation. Five completed the full survey; two left interviews early due to clinical duties. The group included two simulation trainers and five residents, aged 28 to 63, none of whom had prior VR experience. Participant details are shown in Table 2.

**Table 2:** Demographics.

Participants	Interview	Survey	Gender	Age	Role	Years at LUMC	VR Experience	
							Private	Professional
P1	x	x	Female	28	Resident	1	–	–
P2	x		Female		Resident			
P3	x	x	Female	33	Resident	4	–	–
P4	x	x	Male	33	Resident	6	–	–
P5	x	x	Male	38	Trainer	10	–	–
P6	x	x	Male	63	Trainer	30	–	–
P7	x		Female		Resident			

### 3.2 User experience

#### 3.2.1 Quantitative results

The quantitative analysis of the survey data revealed that the overall scores for presence, agency and perceived value of the VR training was moderate to moderately high. Physical presence had the highest mean score *M* = 3.64 (SD = 0.661) while self-presence scores were slightly below the midpoint of the scale *M =* 2.44 (SD = 1.09) suggesting that participants had a fairly high sense of really being present in an operating room. However, they did not quite feel that their virtual presence was an extension of their real body. Agency scores were moderately high *M =* 4.85 (SD = 0.945), indicating that participants felt some control over their movements in the virtual environment. Participants scored slightly below the midpoint regarding the experience of adverse events such as nausea, disorientation and dizziness *M =* 2.93 (SD = 0.894). When asked about the value of the VR training in comparison to physical simulation training with a mannequin, participant' scores were slightly above the midpoint level of the scale *M =* 3.20 (SD = 0.945). Table 3 shows an overview of the quantitative outcomes.

**Table 3:** Descriptive Statistics.

	N	Range	Minimum	Maximum	Mean	Std. Deviation
MPS total*	5	1.67	1.93	3.60	3.04	0.661
Physical presence*	5	1.00	3.20	4.20	3.64	0.456
Social presence*	5	2.60	1.40	4.00	3.04	1.090
Self presence*	5	2.20	1.20	3.40	2.44	0.817
Agency**	5	2.25	3.50	5.75	4.85	0.945
Physical discomfort*	5	2.00	2.00	4.00	2.93	0.894
Perceived Value VR Training*	5	2.50	1.50	4.00	3.20	1.037

*Notes:* * measured on a 5-point Likerts scale; ** measured on a 7-point Likert scale,

#### 3.2.2 Qualitative results

The qualitative analysis of the observational data and interview data provides more contextual information for these quantitative scores and more in-depth information about the user experience and first reaction of towards the VR-scenario training. These insights will be discussed in the following paragraphs.

#### 3.2.3 General user experience

Most participants described the VR-scenario training as an enjoyable experience. They valued the realistic design of the operating room and medical equipment in the virtual environment. However, most participants, described the interaction with the controllers as challenging and something to get used to. As one participant noted:

*“I'm not a gamer, so I really can't manage those hand controls.”* (P6).

Three participants reported mild physical discomfort including nausea and disorientation. One participant indicated that this feeling mostly subsided once she got used to the virtual environment. None of the participants experienced continuous discomfort or had to stop the experience. Some participants also commented on difficulties adjusting the VR glasses which led to visibility issues during the simulation. Other technical issues that were noticed included objects “jumping” away and the ability to walk into objects or other avatars in the virtual environment.

#### 3.2.4 Look and feel of the virtual environment & physical presence

Participants were positive about the accuracy and detail of the virtual operating room and medical equipment. When discussing this topic one of the residents said:

*“Yes, it looked amazing! Really… That cart actually looked just like ours – it was funny to see. That was really well done.”* (P1).

However, some aspects such as the size and placements of certain objects was perceived as slightly inaccurate. They also noted that some equipment was missing in the current prototype. Despite these inaccuracies participants experienced a fairly high sense of physical presence which is in line with the survey outcomes. One of the trainers described the experience as follows:

*“Yes, amazing! I really felt like I was there.”* (P6).

#### 3.2.5 Functional interactions, agency & self-presence

Although participants praised the realism of the medical interactions, they also experienced high cognitive effort when performing the tasks in the virtual simulation. Most of these interactions felt somewhat unintuitive and challenging to perform in VR. As one of the residents stated:

*You can't actually grab anything. Normally, when placing an IV, you hold the hand, like this… and then insert the needle. But that's not possible here — now you have to use buttons. That felt… different.”*(P4).

Because of the high cognitive load participants paid less attention to the scenario and the other players. As one of the residents put it:

*“When you're focused on picking up the syringe, you're so busy with that, that I actually didn't notice anymore whether she [the patient] was… still alive.”* (P7).

Concerning agency**,** participants felt not completely in control of their actions. They indicated that their actions sometimes did not match their intention and that their movements lacked precision. They were also unsure if a task was performed correctly. When discussing this topic one of the residents stated:

*“You feel like you have a bit less control. You press the button and whoop — it goes really fast! Don't overdose!”* (P1).

Several participants reported that they had difficulties grabbing objects or that objects were unintentionally dropped. This lack of control also influence the feeling of being immersed in the VR-scenario. As expressed by the same resisdent:

*“At one point, I really wanted to connect the pulse oximeter, but the cart kept moving away or something — and you're just like, I just want to grab it! When it doesn't quite work, that's when you really realize you're in a simulation or VR.”* (P1).

Although agency was somewhat limited, the quantitative scores indicated that participants still felt some control of their actions.

When discussing self-presence, participants had a mixed reaction how natural and intuitive their virtual embodiment felt. This is also reflected in the quantitative scores. One of the trainers reported a high degree of self-presence although he found it confusing that only his hands were visible:

*Absolutely, it felt very natural. That's why I was a bit disappointed there weren't any arms attached — because those really felt like my hands.”* (P6).

Another participant stated that being able to walk around without teleporting would benefit the experience of self-presence:

*The one thing I still think would be useful is being able to actually walk. Right now, you teleport of course, and that still feels a bit… off. I mean, eventually it worked, but I think if you could walk, you'd feel more like, ‘I need to get there quickly,’ you know?”* (P1).

The limitations that were experienced while interacting in the virtual environment can be partially attributed the fact that participants had no previous experience with VR and had to still get used to the controllers while being in the simulation. The majority of participants were confident that with more practice interactions would become more intuitive and that their sense of agency and self-presence would increase.

#### 3.2.6 Social presence and collaboration

Participants generally reported that they were aware of the other users while they were in the simulation. They valued that they could interact with the other users and tried out small gestures like waving. Verbal communication was possible and became more natural as participants grew more familiar with the VR controllers. However some participants indicated that they were so focused on their own tasks that they paid little attention to the others. Moreover participants could not recognize each as they were represented as abstract dummy avatars without names of role description. As one of the trainers highlighted:

*“I was aware of my own hands, but sometimes I saw other hands without seeing a person attached to them. I really missed the realistic presence of the person.”* (P6).

Another limitation to better collaboration was the lack of peripheral vision due to the VR glasses. Participants had to deliberately turn their head to see each other. Additionally, participants experienced a audio-visual mismatch: sometime a voice of another user seemed distant while the avatar appeared nearby.

With regard to the social presence of the patient avatar participants valued the realism that is not possible with the traditional mannequins like changes in skin colour with low blood saturation. However, participants noted that the patient avatar was still in cloth which is not usually the case in the given scenario.

This mixed experiences regarding social presence are in line with the quantitative score.

#### 3.2.7 Value for non-technical skills training and future application

Participants view the VR-scenario training as a valuable learning tool for training non-technical skills in a simulated setting. Advantages that were mentioned included the possibility to simulate complex scenario's with a high degree of realism. As highlighted by one of the residents:

*“I think you can simulate so much more with this than with a mannequin. With a mannequin, you're physically in the same room, but someone has to tell you, ‘the mannequin is turning blue.’ In VR, you can actually make the patient turn blue. That makes it feel much more realistic, and especially for CRM and communication, people might really start to feel stressed. And from that, you can learn a lot — how you communicate and how you act.”* (P1).

Participants also valued the multiplayer feature that allows them to practice together. The option to practice remotely was also viewed as an advantage. However, participants emphasized that residents need time to get familiar with the controllers and practice the interactions before the training could be used effectively.

#### 3.2.8 Recommendations

The participants had several recommendations to further improve the current prototype and the simulation experience:

1. **Practice interactions:** First and foremost, as already became clear in the previous paragraphs, users of the simulation training need the opportunity to practice and get familiar with the controllers and interaction before engaging in a complex scenario training.
2. **Limit teleportation:** Although teleportation was one of the more intuitive control features, limiting it would help to increase the feeling of presence and spatial orientation during practice.
3. **Feedback:** Participants highlighted the need for some form of feedback to confirm that a task is performed correctly, especially given the lack of tactile cues.
4. **Design of the users' avatars:** Participants preferred a more realistic, human-like representations of their avatar en recommended including the users' names to help them recognize each other.
5. **Add other medical interactions:** To expand the scope of the current prototype participants recommended to expand the current medical interactions and add more, such as intubation.
6. **Seamless communication:** Seamless communication was perceived as key to make the VR-scenario applicable for team training. Any delay in the audio transmission would affect the collaboration and realism of the training.
7. **Remote or on location training:** Regarding the future application of the VR-scenario training, Participants could imagine using the simulation both remotely and on-site.

## 4 Discussion and conclusion

### 4.1 Discussion

This study explored the user experience and potential value of a VR-scenario for anaesthesiologists in training focusing on non-technical skills such as teamwork, leadership and communication. Our approach was in line with Kirkpatrick's first evaluation level (reaction). For our study we used a combination of qualitative and quantitative approaches to provide a broad perspective on user experience.

Overall participants described the VR-scenario as a unique and enjoyable learning experience. Participants valued the realism of the operating room and the medical equipment which was reflected in the moderately high sense of physical presence. The high degree of realism gave them the sense of really being in an actual operating room. Only minor levels of physical discomfort were reported. These results suggest that the VR environment was effective in creating a realistic and engaging environment for the scenario training.

Although the quantitative agency scores were also moderately high, qualitative results revealed that agency and self-presence was negatively affected by the interaction design and difficulties with the VR controllers. For many, the interaction design was unintuitive and imposed a steep learning curve, particularly for participants without prior gaming or VR experience. Users reported a high cognitive load while performing medical tasks which sometimes lead to reduced situational awareness and difficulties in collaborating with other team members and attending to the patient cues. Participants also missed some form of feedback on their task performance. These findings suggest that for future development work, we need reiterate on how to transfer fine-motoric medical tasks into intuitive VR interactions and on how to provide feedback without tactile cues. In addition, an onboarding environment should be added to the simulation, where users can familiarise themselves with the VR controllers and practice medical tasks before engaging in the scenario training.

The sense of social presence and the reaction to collaborating with other users was also mixed. Although participants valued the multiplayer function and saw great potential for team training collaboration was currently hindered by the abstract avatar design, limited peripheral vision and occasional audio-visual mismatch. The results suggest that for future development work we need to reiterate on the multiplayer feature and create more alignment between auditive and visual cues and improve the design of the user's avatars.

Despite these challenges, participants expressed enthusiasm about the value and future potential of the VR training for developing non-technical skills such as communication and teamwork. Several participants indicated that repeated exposure and an initial familiarisation phase would enhance the effectiveness of VR-scenarios in educational settings. Moreover, the multiplayer functionality, the opportunity to simulate complex team-based scenarios with a high degree of realism and the opportunity to practice remotely or on-site were perceived as important strengths of the VR approach.

From an internal validity perspective, several limitations must be considered. The relatively small sample size of seven participants restricts the generalizability of the findings. Furthermore, the participants may not fully represent the diversity of backgrounds and levels of experience present in the broader population of anaesthesiologists. The reliance on self-reported measures introduces the potential for response bias, particularly given the novelty of VR technology. Nevertheless, the use of concomitant qualitative and quantitative data strengthens the study by offering both quantitative benchmarks and contextualized feedback on user experiences.

When placing these findings in the context of the broader VR literature in medical simulation, our results are largely consistent with previous research. Many VR-based medical training studies report high engagement levels but identify usability challenges, particularly related to interface design and physical side effects such as cybersickness [[20], [21], [22]]. The emphasis on VR's potential for non-technical skills training resonates with growing calls in the literature for simulation tools that do more than rehearse technical procedures, instead fostering communication, leadership, and teamwork under realistic pressures [23].

The creation of innovative solutions to unprecedented problems amidst unexpected circumstances necessitates a substantial adaptive expertise, which refers to a system's aptitude to adapt to intricate, dynamic, and demanding conditions. Clinical teams encounter significant challenges in exercising adaptive expertise during periods of elevated stress.

### 4.2 Innovation

This study introduces innovation in both methodological approach and educational application. By explicitly applying Kirkpatrick's evaluation model to the early assessment of a VR-based simulation for anaesthesiology, it addresses a gap in the literature where structured evaluation frameworks are often lacking [23,24]. The focus on user reaction (Level 1) provides a foundation for systematic future evaluations targeting learning, behaviour change, and organizational outcomes.

Moreover, the integration of quantitative (VEQ and MPS scales) and qualitative thematic analysis offers a novel, comprehensive perspective on user experience that goes beyond simple satisfaction surveys alone. Previous reviews have identified a predominance of single-method evaluations in VR simulation studies, often focused narrowly on usability or enjoyment without deeper analysis of presence and interaction quality [20,24].

Finally, this study highlights VR's potential not only for technical skill rehearsal, but for fostering non-technical skills such as teamwork and communication—areas increasingly recognized as critical for clinical performance under pressure. In doing so, it supports the translation of VR simulation methods into non-technical, team-based training, promoting the development of adaptive expertise in clinical teams working in dynamic, high-stress environments.

### 4.3 Conclusion

This study demonstrates that VR-based simulation offers a promising tool for non-technical skills training in anaesthesiology, providing an engaging and realistic learning environment. While users appreciated the immersive design and collaborative potential, challenges related to interaction design, physical comfort, and technical reliability were identified. Addressing these usability aspects will be crucial for maximizing the educational impact of VR simulations. Future studies should expand on these findings by exploring learning outcomes and behavioural changes across larger and more diverse participant groups evaluating higher levels of effectiveness.

## CRediT authorship contribution statement

**Krista Hoek:** Writing – review & editing, Writing – original draft, Visualization, Validation, Methodology, Investigation, Funding acquisition, Formal analysis, Conceptualization. **Monique van Velzen:** Writing – review & editing, Project administration, Conceptualization. **Elise Sarton:** Project administration, Methodology, Conceptualization. **Christina Jaschinski:** Writing – review & editing, Writing – original draft, Visualization, Validation, Project administration, Methodology, Investigation, Funding acquisition, Formal analysis, Conceptualization.

## Declaration of competing interest

Christina Jaschinksi reports financial support was provided by Tech For Future. If there are other authors, they declare that they have no known competing financial interests or personal relationships that could have appeared to influence the work reported in this paper.

## Footnotes

1 TechForFuture is the Centre of Expertise for applied research in High Tech Systems and Materials (HTSM), https://techforfuture.nl/
